# Impact of Intraoperative Opioid Use and a Combined Anesthesia Regimen in Patients Undergoing Radical Prostatectomy for Prostate Cancer in a Single-Center Cohort

**DOI:** 10.3390/jcm13247506

**Published:** 2024-12-10

**Authors:** Julian Marcon, Robert Bischoff, Kaspar Rattenhuber, Michael Chaloupka, Darjusch Askari, Jan-Friedrich Jokisch, Armin J. Becker, Paulo L. Pfitzinger, Patrick Keller, Elena Berg, Christian G. Stief, Daniel Siegl, Christian Kowalski, Alexander Buchner, Nikolaos Pyrgidis, Philipp Weinhold

**Affiliations:** 1 Department of Urology, University Hospital of the LMU Munich, 81377 Munich, Germany; julian.marcon@med.uni-muenchen.de (J.M.); robert.bischoff@med.uni-muenchen.de (R.B.); kaspar.rattenhuber@googlemail.com (K.R.); michael.chaloupka@med.uni-muenchen.de (M.C.); darjusch.askari@med.uni-muenchen.de (D.A.); friedrich.jokisch@med.uni-muenchen.de (J.-F.J.); armin.becker@med.uni-muenchen.de (A.J.B.); paulo.pfitzinger@med.uni-muenchen.de (P.L.P.); patrick.keller@med.uni-muenchen.de (P.K.); elena.berg@med.uni-muenchen.de (E.B.); christian.stief@med.uni-muenchen.de (C.G.S.); philipp.weinhold@med.uni-muenchen.de (P.W.); 2 Department of Anesthesiology, University Hospital of the LMU Munich, 81377 Munich, Germany; daniel.siegl@med.uni-muenchen.de (D.S.); christian.kowalski@med.uni-muenchen.de (C.K.)

**Keywords:** prostate cancer, prostatectomy, overall survival, opioids

## Abstract

**Introduction:** Higher intraoperative opioid doses may be associated with worse long-term oncological outcomes after radical prostatectomy (RP) for prostate cancer. We aimed to evaluate the impact of higher doses of intraoperative opioids and type of anesthesia on biochemical recurrence (BCR) and mortality after RP in a high-volume tertiary center. **Methods:** All patients underwent RP at our center between 2015 and 2021. The role of major intraoperative opioid agents, such as sufentanil remifentanil, and morphine milligram equivalents (MMEs), as well as the type of anesthesia [total intravenous anesthesia (TIVA), versus a combination of TIVA and epidural anesthesia, versus solely epidural anesthesia], was assessed in predicting BCR and survival after RP. **Results:** A total of 1137 patients who had a median age of 66 years (interquartile range: 61–72) were included. Overall, 1062 (93%) patients received TIVA, 37 (3%) received TIVA and epidural anesthesia, and 41 (4%) only epidural anesthesia. At a median follow-up of 431 days (interquartile range: 381–639) from RP, 257 (24%) patients developed a BCR. Accordingly, at a median follow-up of 500 days (interquartile range: 450–750), 33 (2.9%) patients died. The type of anesthesia, as well as the dosage or type of the selected intraoperative opioid agents, did not affect either BCR or long-term overall survival. **Conclusions:** These findings suggest that intraoperative opioid application during RP has no negative oncological impact in the short and long term in patients with localized prostate cancer. Accordingly, combined TIVA and epidural anesthesia, as well as solely epidural anesthesia were associated with similar short- and long-term outcomes compared to TIVA.

## 1. Introduction

Localized prostate cancer (PC) is considered the commonest non-cutaneous malignancy in men, and is typically managed with radical prostatectomy (RP) [1]. Despite the recent advances in surgical techniques and perioperative care, RP is still associated with postoperative complications, which may negatively affect both short-term recovery and long-term oncological outcomes [2]. RP is also often associated with significant perioperative pain, which is commonly managed with intravenous and/or epidural anesthesia [3]. Accordingly, RP can be performed in some rare cases solely with epidural anesthesia [4]. Perioperative anesthesia often includes the use of opioids to reduce pain effectively [5].

Even though opioids are considered a major component of modern anesthetic practice, they might increase perioperative complications [6]. Moreover, accumulating evidence suggests that higher intraoperative opioid doses may be associated with worse long-term oncological outcomes in various malignancies, including PC [7]. It has been postulated that increased opioid use during major abdominal surgery could negatively affect disease-free and overall survival, possibly due to the immunomodulatory effects of opioids on tumor cells [8]. In particular, this effect is hypothesized to be mediated through µ-opioid receptors on tumor cells, which may promote tumor angiogenesis and suppress the immune response [9]. Opioids also modulate immune system function, either by suppressing the immune response to facilitate tumor dissemination or by directly enabling tumor growth and metastasis [10]. To complicate things further, other studies have found no significant impact of intraoperative opioid use on cancer outcomes, indicating a complex and not fully understood relationship between opioid administration and cancer prognosis [11].

Considering the discordance in the available body of literature, it is mandatory to further assess the potential risks that may emerge after intraoperative opioid use during RP. In this context, the present study aims to evaluate the impact of higher doses of intraoperative opioids, particularly modern agents such as remifentanil and sufentanil, on biochemical recurrence (BCR) and mortality after RP in a high-volume tertiary PC center. Moreover, we assess the effect of total intravenous anesthesia (TIVA) versus a combination of TIVA and epidural anesthesia, as well as versus solely epidural anesthesia.

## 2. Materials and Methods

### 2.1. Study Design

After obtaining approval from our institutional review board, we queried the prospectively maintained database of our tertiary PC reference center for patients undergoing RP between 2015 and 2021. This study conformed with the Declaration of Helsinki, its findings were reported based on the STROBE statement for cohort studies [12], and all included patients provided written informed consent. For this study, we included all patients with PC undergoing RP with complete demographic and oncological data, complete information on the type and dose of perioperatively administered opioids, as well as comprehensive follow-up information. On the other hand, we excluded patients with metastatic PC, positive lymph nodes, those who received neoadjuvant or adjuvant therapies (radiotherapy, androgen-deprivation therapy, or other treatments) for PC, or those with PSA persistence after RP (defined as ≥0.1 ng/mL). Accordingly, we excluded all patients with no follow-up data or with missing data.

### 2.2. Onco-Anesthesia Protocol

In all cases, sufentanil or remifentanil were used during open or minimally invasive RP as intraoperative opioid agents. For the purposes of the analysis, their total intraoperative dosage was calculated. In particular, intraoperative sufentanil and remifentanil doses were standardized to oral morphine milligram equivalents (MMEs), calculated as a continuous variable, and reported per 10 MME. According to current evidence, 10 MME corresponds to 50 μg of intravenous remifentanil, which demonstrates a similar efficacy to that of fentanyl. Accordingly, 10 MME was also considered equivalent to 5 μg intravenous sufentanil, which is about ten times more effective than fentanyl [13]. Moreover, a further subclassification of patients was performed based on the 75th percentile of sufentanil, remifentanil, and 10 MME. More specifically, patients were classified into low- (below 75th percentile), and high- (above 75th percentile) dose recipients of sufentanil, remifentanil, and 10 MME. For patients who required combined epidural anesthesia and TIVA or solely epidural anesthesia, an epidural catheter was placed between T12 and L2, based on the discretion of the anesthesiologist.

### 2.3. Outcomes and Statistics

The primary outcome of the present analysis was to explore the role of major intraoperative opioid agents and type of anesthesia (TIVA versus TIVA + additional epidural anesthesia versus solely epidural anesthesia) in predicting long-term survival and BCR after RP for PC. PSA monitoring after RP was performed every three months during the first year postoperatively, every six months until the third year postoperatively, and annually thereafter. BCR was defined as a rising PSA of ≥0.2 ng/mL on two consecutive measurements. Secondary outcomes assessed the influence of intraoperative opioid dosing on the outcomes of interest, with sufentanil, remifentanil, and 10 MME analyzed individually.

Continuous variables were summarized as medians with interquartile ranges (IQRs) and compared using the Kruskal–Wallis test, while categorical variables were presented as frequencies with proportions and analyzed with the chi-squared test. A univariate Cox regression analysis was conducted to evaluate the impact of intraoperative opioid use and anesthesia type on time-to-biochemical-recurrence (BCR) and long-term overall survival. For all analyses, hazard ratios (HRs) with the corresponding 95% confidence intervals (CIs) were computed. All analyses were performed with the R statistical software (version 3.6.3, R Core Team 2020) and a two-sided *p*-value < 0.05 was considered statistically significant.

## 3. Results

### 3.1. Baseline Characteristics

A total of 1137 patients underwent RP for localized PC at our institution between 2015 and 2021, and fulfilled the selection criteria. Their median age was 66 years (IQR: 61–72), and their preoperative PSA values 9 ng/mL (IQR: 6–15). A robotic RP was performed in 445 (39%) cases. The median time of operation was 107 min (IQR: 71–172) and the median loss of blood was 200 mL (IQR: 100–400). Overall, 515 (45%) patients had at least a pT3 tumor, and 359 (32%) patients were diagnosed with positive surgical margins, based on the pathological findings. Their postoperative Gleason score was 6 in 105 (9%) cases, 7a in 398 (35%), 7b in 275 (24%), and above 8 in 359 (32%).

Overall, 1062 (93%) patients received TIVA, 37 (3%) received TIVA and epidural anesthesia, and 41 (4%) only epidural anesthesia. The median dosage of sufentanil was 10 µg (IQR: 10–15), the median dosage of remifentanil was 2972 µg (IQR: 957–4449), and the median dosage of 10 MME was 18 µg (IQR: 2–73). A high dose was defined as the 75th percentile or above, for each opioid: 15 µg for sufentanil, 4449 µg for remifentanil, and 73 µg for 10 MME. Patients receiving TIVA required a lower dosage of sufentanil and a higher dosage of remifentanil (*p* < 0.001), compared to patients receiving TIVA and epidural anesthesia or only epidural anesthesia. Furthermore, the length of hospital stay did not differ among the three groups (*p* = 0.3). Table 1 provides an overview of the baseline characteristics for the entire study cohort, along with comparisons stratified by the type of anesthesia.

### 3.2. Time to BCR

**Figure 1:**
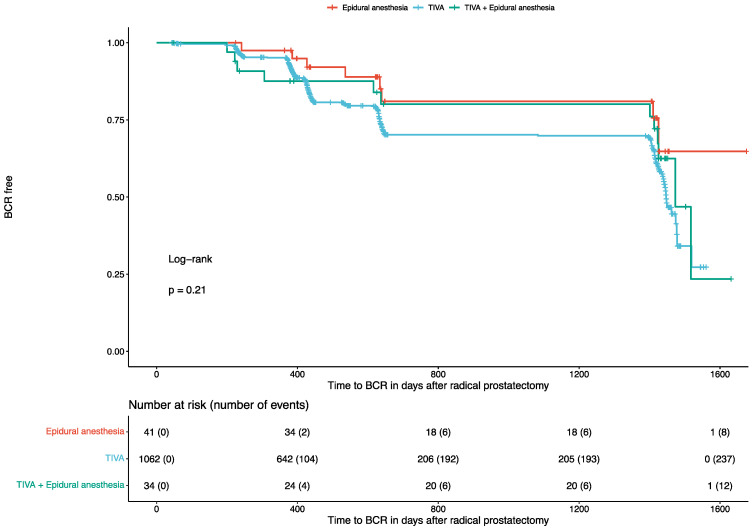
Kaplan–Maier curve for BCR in patients undergoing radical prostatectomy. BCR: Biochemical Recurrence.

After a median follow-up of 431 days (IQR: 381-639) from RP, 257 (24%) patients developed a BCR. Of them, 237 (22%) were reported in patients undergoing TIVA, 12 (35%) in patients receiving TIVA and epidural anesthesia, and 8 (20%) in patients receiving only epidural anesthesia. In the univariate Cox regression analysis with respect to the time to BCR, TIVA was not associated with better outcomes (HR: 0.81, 95% CI: 0.45 to 1.46, *p* = 0.5) compared to combined TIVA and epidural anesthesia. Similarly, TIVA was not associated with better outcomes (HR: 0.55, 95% CI: 0.27 to 1.12, *p* = 0.1) compared to solely epidural anesthesia. The corresponding Kaplan–Meier curve (log-rank test: *p* = 0.21) is displayed in Figure 1. Accordingly, patients who received higher intraoperative doses of remifentanil (*p* = 0.9), sufentanil (*p* = 0.3), or MME (*p* = 0.4) also presented similar BCR rates. The results of the univariate Cox regression analysis for the time-to-BCR are available in Table 2.

### 3.3. Overall Survival

**Figure 2:**
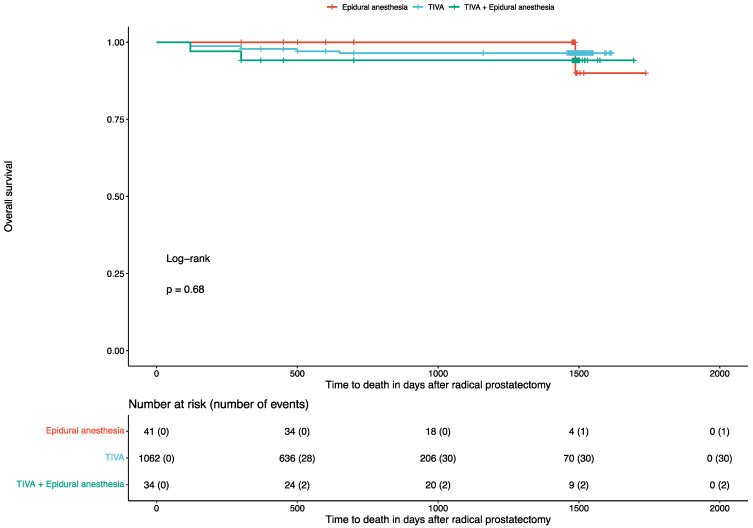
Kaplan–Maier curve for overall survival in patients undergoing radical prostatectomy.

After a median follow-up of 500 days (IQR: 450–750) from RP, 33 (2.9%) patients died. Of these, 30 (2.8%) were reported to be patients undergoing TIVA, 2 (5.9%) to be patients receiving TIVA and epidural anesthesia, and 1 (2.4%) to be a patient receiving only epidural anesthesia. In the univariate Cox regression analysis with respect to the time to death, TIVA was not associated with better outcomes (HR: 1.82, 95% CI: 0.43 to 7.66, *p* = 0.4) compared to combined TIVA and epidural anesthesia. Similarly, TIVA was not associated with better outcomes (HR: 0.75, 95% CI: 0.1 to 5.51, *p* = 0.8) compared to solely epidural anesthesia. The corresponding Kaplan–Meier curve (log-rank test: *p* = 0.68) is displayed in Figure 2. Accordingly, patients who received higher intraoperative doses of remifentanil (*p* = 0.8), sufentanil (*p* = 0.8), or MME (*p* = 0.4) also presented similar survival rates. The results of the univariate Cox regression analysis for the time-to-death are available in Table 2.

## 4. Discussion

The results of this cohort study involving patients undergoing RP for PC suggest that neither the type nor the dosage of intraoperative opioid agents appears to influence BCR rates or long-term overall survival. Based on the previous notion, patients who required a higher dosage of sufentanil, remifentanil, or 10 MME also presented similar outcomes. Accordingly, it seems that the combination of TIVA and epidural anesthesia, as well as the use solely of epidural anesthesia, lead to similar BCR rates and overall survival compared to TIVA.

Opioids play a key role in perioperative anesthesia and pain management for cancer surgeries, including RP. The role of opioids extends beyond pain relief, as they have complex effects on immune function and, potentially, on cancer progression [14]. Preclinical studies indicate that opioids may exert immunosuppressive effects, which may lead to enhanced tumor growth and micro-metastases through impaired immune response [15]. However, conflicting evidence on the matter exists, with some animal models suggesting a potential antitumor role of opioids, indicating the complexity of opioid effects in cancer settings [16]. To complicate things further, our analysis indicates that the type of anesthesia, as well as higher intraoperative opioid doses, do not negatively affect BCR and overall survival after RP.

It should be stressed that our findings are in line with previous studies on the matter. Studies comparing TIVA versus combined general and epidural anesthesia suggest that it is safe and effective in terms of perioperative and long-term outcomes [4]. Accordingly, studies focusing solely on epidural anesthesia indicate that it may be performed with similar outcomes, compared to TIVA. Nevertheless, the latter should be predominantly performed in high-volume centers specializing in RP, to guarantee lower intraoperative complication rates [17]. These centers benefit from experienced surgical and anesthetic teams capable of optimizing anesthesia strategies, including balanced use of intravenous and epidural opioids, as part of a multimodal pain management approach [18]. Based on the previous notion, the present study deriving from an excellence center for PC demonstrated that the operative time of RP was short, and the total blood loss was minimal. Interestingly, for all robotic prostatectomies, a TIVA was preferred.

The present cohort study consists of patients requiring RP due to aggressive PC. Notably, nearly half of the patient population presented with locally advanced disease, and a significant proportion had high Gleason scores, indicating a more aggressive tumor profile [19]. These factors contributed to higher rates of positive surgical margins when compared to other contemporary cohort studies, where patient profiles might reflect less advanced disease [20]. The latter is also reflected by the fact that most patients were operated on with an open approach. Consequently, approximately one-fourth of the patients experienced BCR, highlighting the challenging nature of managing high-risk PC cases in this cohort [21]. This underlines the need for tailored therapeutic strategies and rigorous follow-up in similar patient populations [22]. Still, the combination of intraoperative opioid agents, as well as the selected protocol of anesthesia, do not seem to affect short- and long-term outcomes.

Despite the holistic nature of this study, further research is mandatory to deepen our understanding of the relationship between intraoperative opioid use, anesthesia techniques, and oncological outcomes after RP. Future research should focus on multicenter, prospective cohort studies, or randomized controlled trials, to minimize potential biases and ensure the generalizability of our findings. These studies should incorporate more detailed assessments of the immunological and molecular mechanisms underlying opioid–tumor interactions, including the role of µ-opioid receptors and their impact on tumor microenvironments. Additionally, comparative analyses of novel analgesic protocols, including opioid-sparing regimens, regional anesthesia techniques, and multimodal pain-management approaches, are warranted to identify optimal strategies that balance effective pain control with minimized risks of perioperative and long-term complications. Moreover, big-data analysis may enable the identification of specific patient subgroups most susceptible to variations in anesthesia protocols or opioid dosages, thus paving the way for personalized anesthetic strategies. Finally, evaluating the impact of emerging surgical techniques, such as robotic-assisted RP, in conjunction with modern anesthesia protocols, will provide valuable insights into improving patient outcomes.

It should be acknowledged that, in the present study, we did not provide an opioid-free group. However, the current body of evidence suggests that the focus should not necessarily be on the complete elimination of opioids, but rather on their reduction and more differentiated use. A balanced anesthesia, with validated concepts of multimodal, opioid-based analgesia, continues to hold significant value and remains the current standard of care [23]. Indeed, an opioid-free anesthesia approach is uncommon in Germany and the Western world overall [24]. In our center, and, to the best of our knowledge, in any other center in Germany, RP is performed with intraoperative opioid administration [25]. Therefore, an opioid-free approach is not supported by strong evidence, and represents a significant deviation from current standards of care [26]. Including an opioid-free arm would have required substantial structural changes and may not reflect real-world practices. Nonetheless, future studies designed to include an opioid-free group would provide further insights into the impact of opioids on complications and oncological outcomes.

It should be underlined that the findings of the present study were tempered by some important limitations relevant to its single-center design. In order to provide homogenous outcomes, we restricted our analyses to patients with non-metastatic PC undergoing RP. Therefore, the role of anesthesia in predicting long-term survival and BCR in patients requiring neoadjuvant or adjuvant treatments could not be assessed. It should also be acknowledged that the three groups were not evenly distributed, with most of the included patients receiving only TIVA. While this reflects routine clinical practice, it may have introduced a potential limitation that could complicate the interpretation of our findings. Importantly, we could not evaluate the role of major intraoperative opioid agents and type of anesthesia on perioperative and long-term complications such as transfusion, ileus, sepsis, cardiorespiratory complications, renal impairment, admission to the intensive care unit, or reintervention rates. Similarly, no information on complication rates based on the Clavien–Dindo classification could be provided [27]. Still, it should be noted that perioperative and long-term complications after RP occur rarely [28,29]. Of note, the impact of other intraoperative or perioperative analgesics, including additional local anesthetics, non-steroidal anti-inflammatory drugs, or metamizole, on both short- and long-term outcomes could not be determined. Moreover, given that both the BCR and the death rates were relatively low, we could not adjust for further risk factors and comorbidities through a multivariable Cox regression analysis. It was also beyond the scope of the present study to assess for cancer-specific survival. Still, patients harboring PC have worse overall survival than cancer-specific survival [30,31]. This should be stated clearly. Finally, considering that we selected only patients who underwent RP in the last ten years, we could not explore the role of the evolution of different surgical techniques and perioperative medical care on BCR and survival.

## 5. Conclusions

The present analysis indicates that intraoperative opioid application during RP has no negative impact on prognosis in patients with localized prostate cancer. Accordingly, combined TIVA and epidural anesthesia, as well as solely epidural anesthesia, were associated with similar short- and long-term outcomes compared to TIVA. Nevertheless, further high-quality studies are mandatory to corroborate our findings.

## Figures and Tables

**Table 1:** Baseline characteristics of patients undergoing radical prostatectomy based on the type of anesthesia. Values are presented as median (interquartile range) or n (%). MME: Morphine Milligram Equivalent.

Characteristic	Overall, n = 1137	TIVA, n = 1062	TIVA + Epidural Anesthesia, n = 34	Epidural Anesthesia, n = 41	*p*-Value
Age (years)	66 (61–72)	66 (61–72)	68 (64–71)	65 (62–70)	0.8
Preoperative PSA (ng/mL)	9 (6–15)	9 (6–15)	8 (5–12)	8 (5–15)	0.8
Robotic prostatectomy	445 (39%)	445 (42%)	0 (0%)	0 (0%)	<0.001
Operative time (minutes)	107 (71–172)	114 (72–175)	103 (69–129)	67 (62–76)	<0.001
Blood loss (mL)	200 (100–400)	200 (100–400)	300 (225–600)	200 (100–300)	0.016
T after prostatectomy					0.011
T2	625 (55%)	597 (56%)	12 (35%)	16 (39%)	
T3	505 (44%)	459 (43%)	21 (62%)	25 (61%)	
T4	7 (0.6%)	6 (0.6%)	1 (2.9%)	0 (0%)	
Gleason score					0.8
6	105 (9.2%)	98 (9.2%)	3 (8.8%)	4 (9.8%)	
7a	398 (35%)	379 (36%)	9 (26%)	10 (24%)	
7b	275 (24%)	257 (24%)	9 (26%)	9 (22%)	
8	142 (12%)	129 (12%)	6 (18%)	7 (17%)	
9	200 (18%)	183 (17%)	7 (21%)	10 (24%)	
10	17 (1.5%)	16 (1.5%)	0 (0%)	1 (2.4%)	
Positive surgical margins	359 (32%)	329 (31%)	15 (44%)	15 (37%)	0.2
Length of hospital stay (days)	8 (8–9)	8 (8–9)	8 (7–9)	9 (8–9)	0.3
Sufentanil (µg)	10 (10–15)	10 (10–10)	23 (10–30)	5 (5–9)	<0.001
Remifentanil (µg)	2972 (957–4449)	3021 (980–4518)	840 (680–1690)	178 (92–212)	<0.001
10 MME	18 (2–73)	19 (2–74)	6 (3–15)	1 (1–3)	<0.001

**Table 2:** Univariate Cox regression analysis for BCR and overall survival in patients undergoing radical prostatectomy. BCR: Biochemical Recurrence, CI: Confidence Interval, HR: Hazard Ratio, MME: Morphine Milligram Equivalent.

	Outcome	Univariate Cox Regression		
		HR	95% CI	*p*-Value
BCR	Type of anesthesia			
	TIVA	—	—	
	TIVA + Epidural anesthesia	0.81	0.45, 1.46	0.5
	Epidural anesthesia	0.55	0.27, 1.12	0.10
	High dose of remifentanil	1.03	0.70, 1.52	0.9
	High dose of sufentanil	0.86	0.64, 1.15	0.3
	High dose of MME	1.00	0.99, 1.01	0.4
Mortality	Type of anesthesia			
	TIVA	—	—	
	TIVA + Epidural anesthesia	1.82	0.43, 7.66	0.4
	Epidural anesthesia	0.75	0.10, 5.51	0.8
	High dose of remifentanil	1.12	0.40, 3.14	0.8
	High dose of sufentanil	0.92	0.41, 2.03	0.8
	High dose of MME	1.00	0.99, 1.01	0.4

## Data Availability

All data generated or analyzed during this study are included in this article. Further inquiries can be directed to the corresponding author.
